# A Polyphenol Rich Extract from *Solanum melongena* L. DR2 Peel Exhibits Antioxidant Properties and Anti-Herpes Simplex Virus Type 1 Activity In Vitro

**DOI:** 10.3390/molecules23082066

**Published:** 2018-08-17

**Authors:** Antonella Di Sotto, Silvia Di Giacomo, Donatella Amatore, Marcello Locatelli, Annabella Vitalone, Chiara Toniolo, Giuseppe Leonardo Rotino, Roberto Lo Scalzo, Anna Teresa Palamara, Maria Elena Marcocci, Lucia Nencioni

**Affiliations:** 1 Department of Physiology and Pharmacology “V. Erspamer”, Sapienza University, P.le Aldo Moro 5, 00185 Rome, Italy; silvia.digiacomo@uniroma1.it; 2 Department of Public Health and Infectious Diseases, Istituto Pasteur Italia-Fondazione Cenci-Bolognetti, Sapienza University, P.le Aldo Moro 5, 00185 Rome, Italy; donatella.amatore@uniroma1.it (D.A.); annateresa.palamara@uniroma1.it (A.T.P.); mariaelena.marcocci@uniroma1.it (M.E.M.); 3 Department of Pharmacy, University “G. D’Annunzio” of Chieti-Pescara, Via dei Vestini 31, 66100 Chieti, Italy; m.locatelli@unich.it; 4 Department of Environmental Biology, Sapienza University, P.le Aldo Moro 5, 00185 Rome, Italy; chiara.toniolo@uniroma1.it; 5 Research Centre for Genomics and Bioinformatics (CREA-GB), Via Paullese 28, Lodi, 26836 Montanaso Lombardo, Italy; giuseppeleonardo.rotino@crea.gov.it; 6 Research Centre for Engineering and Agro-Food Processing (CREA-IT), Via Venezian 26, 20133 Milan, Italy; roberto.loscalzo@crea.gov.it

**Keywords:** eggplant peel, polyphenols, HSV-1, Warburg effect, antioxidant activity, antiviral agents, vegetable waste

## Abstract

DR2B and DR2C extracts, obtained by ethanolic maceration of peel from commercially and physiologically ripe aubergine berries, were studied for the antioxidative cytoprotective properties and anti-HSV-1 activity, in line with the evidence that several antioxidants can impair viral replication by maintaining reducing conditions in host cells. The antioxidative cytoprotective effects against tBOOH-induced damage were assessed in Caco2 cells, while antiviral activity was studied in Vero cells; polyphenolic fingerprints were characterized by integrated phytochemical methods. Results highlighted different compositions of the extracts, with chlorogenic acid and delphinidin-3-rutinoside as the major constituents; other peculiar phytochemicals were also identified. Both samples reduced reactive oxygen species (ROS) production and exhibited scavenging and chelating properties. DR2C partly counteracted the tBOOH-induced cytotoxicity, with a remarkable lowering of lactate metabolism under both normoxia and hypoxia; interestingly, it increased intracellular GSH levels. Furthermore, DR2C inhibited the HSV-1 replication when added for 24 h after viral adsorption, as also confirmed by the reduction of many viral proteins’ expression. Since DR2C was able to reduce NOX4 expression during HSV-1 infection, its antiviral activity may be correlated to its antioxidant properties. Although further studies are needed to better characterize DR2C activity, the results suggest this extract as a promising new anti-HSV-1 agent.

## 1. Introduction

Medicinal plants have played a central role as source of new bioactive molecules in the years, thus leading to the discovery of more than half of the current pharmaceuticals. Also, they can be used as crude extracts or “standard enriched fractions” in pharmaceutical preparations, or as home-made remedies [1]. The strong interest for natural products is due to several factors, including the need for new, more effective pharmacological agents, the remarkable scaffold diversity and biological activities of natural molecules, and the numerous traditional uses in ethnomedicines, which often support researchers in the selection of a suitable plant for pharmacological study [2]. Particularly, large numbers of plant-derived products, including pure compounds, fractions, and crude extracts, have been approached as possible new antimicrobial strategies against herpes simplex virus type 1 (HSV-1) infections [3,4,5]. HSV-1 is a human enveloped-DNA virus that persists along with the lifetime of the host in sensory neurons, where it establishes latency [6]. Its reactivation induces recurrent infections that cause a broad spectrum of clinical symptoms, varying from mild skin vesicular lesions to severe and rare manifestations, such as encephalitis and keratitis, especially during immune deficit conditions [7]. The administration of the antiviral agent acycloguanosine (acyclovir) is the primary therapeutic approach to inhibit HSV-1 infections, but not to avoid recurrence arising from the reactivation of the latent virus [8,9]. Therefore, looking for new bioactive agents against HSV-1 infections, to be used as alternative or complementary treatments, is emerging as an important requirement for human health.

Recently, polyphenols, isoprenoidal glycosides, phenolics, peptides, benzophenones, and pyranocoumarins have been studied for their potential anti-HSV-1 properties [10,11,12]. Particularly, flavonoids and anthocyanins have been found to possess anti-HSV-1 effects, likely due to antioxidant mechanisms [10,13]. In fact, it is known that viral infections, including HSV-1, are often associated with oxidative stress that is useful for the virus to replicate in cells [14,15,16], and antioxidant molecules, like glutathione (GSH) or its *n*-butanoyl derivative (GSH-C4), by restoring the intracellular redox conditions, are able to inhibit HSV-1 replication [17,18,19].

In line with this evidence, the present study was aimed at evaluating the potential antiherpetic activity of *Solanum melongena* L., commonly known as eggplant, aubergine, or brinjal. Besides the culinary interest, this species has been used for several medicinal purposes in folk medicine, particularly by Asiatic people [20,21]. It is reported to be topically applied for treating several skin diseases, including dermatitis, eczema, sores, infections, and human papilloma virus-caused warts and condylomata [22,23]. Also, glycoalkaloids from eggplant have been reported to counteract free radical damage, thus showing skin-protective power [24]. Several studies also described the health benefits associated with phenolics extracted from eggplant, thus suggesting its possible role as a nutraceutical source [25]. A remarkable diversity in polyphenols was found in this plant, with chlorogenic acid and the anthocyanins delphinidin 3-rutinoside (D3R) and/or nasunin (NAS) as the main types represented, especially in the peel [26,27]. These compounds have been reported to possess a wide spectrum of nutraceutical properties, such as antioxidant, antiproliferative, and anti-inflammatory [28,29,30,31,32]; also, chlorogenic acid has been found to possess multi-antiviral activities [32]. 

Taking into account this evidence, in the present study, we evaluated the potential antioxidative cytoprotective effects and the anti-herpetic activity of the DR2B and DR2C extracts, obtained by ethanolic maceration of the berry peel of *S. melongena* L. DR2 genotype, collected at both commercial (B) and physiological (C) ripening stages, as reported by Mennella et al. [33]. 

Polyphenol fingerprints were characterized by integrated chromatographic (HPTLC and HPLC) and spectrophotometric techniques, in order to evaluate the possible contribution of these compounds to the biological activity of DR2B and DR2C extracts. The antioxidant cytoprotective effects against the oxidative damage induced by *tert*-butylhydroperoxide (tBOOH) were studied in human colon cancer Caco2 cells, which are sensitive to oxidant injury and represent a widely standardized model for studying oxidative stress and metabolic fate of oxidant species [34,35]. In these cells, we also studied if the treatments affected the levels of reactive oxygen species (ROS), content of reduced glutathione (GSH), and cell metabolism as a result of the antioxidant activity. The radical scavenging activity and the iron chelating/reducing properties were assayed too. At last, taking into account the key role of intracellular redox state in regulating viral replication, DR2B and DR2C extracts were also assessed for their potential anti-HSV1 activity in monkey kidney epithelial (Vero) cells, known to be highly permissive to the virus [36].

## 2. Results and Discussion

### 2.1. Phytochemical Analysis

Colorimetric determinations highlighted the presence of total polyphenols and flavonoids in all the samples of DR2 aubergine. Particularly, the amounts of polyphenols and flavonoids were found increased in DR2C with respect to DR2B extract (amount about doubled and tripled for polyphenols and flavonoids respectively) (Table 1).

Eggplant is known to contain high levels of phenolic compounds, mainly phenolic acids (chlorogenic acid, caffeic acid, *p*-coumaric acid) in pulp and anthocyanins, such as NAS and delphinidin conjugates in the peel [37,38]. Total phenolic content has been found varied in different extracts, with acidified ethanol being the most frequently used extraction solvent. Fresh eggplant peels were found to contain high levels of total phenols, with major differences due to the extraction method [39,40]. Lower amounts were reported in the extracts of whole fresh fruits [41] and in those from dried peels [42]. Several factors have been shown to affect the phenolic amount of eggplant, among which, the harvesting period [43,44]. 

Under our experimental conditions, the ratio between the total amounts of polyphenols and flavonoids, calculated as chlorogenic acid equivalents, was about 21.7 and 12.6 for DR2B and DR2C, respectively: this suggests that physiological ripeness markedly increases the flavonoid levels in DR2 eggplant peel. Our results agree with literature that reported flavonoids generally representing about 10–15% of total phenolics in eggplant [45].

On the basis of the drug extract ratio (DER) values obtained for each sample (i.e., DR2B, 23:1; DR2C, 25:1), the total flavonoid amount was estimated to be 140 mg/kg (0.014% *w*/*w*) and 392 mg/kg (0.039 % *w*/*w*) in the raw material from DR2B and DR2C aubergine berries, respectively. According to Peterson and Dwyer [46], who classified the flavonoid concentration in foods as low (0.1–39.9 mg/kg), moderate (40–99.9 mg/kg), and high (>100 mg/kg), the peels of DR2B and DR2C aubergine berries produced samples with high flavonoid contents. Similar results were reported by Ji et al. [47], which found a total flavonoid amount of 116.4 mg/kg of fresh eggplant peel, in spite of undetectable levels in the pulp. Lower amounts of flavonoids and tannins were found in ethanolic extract from dried eggplant peels [40,42].

The HPTLC analysis of DR2B and DR2C extracts revealed the presence of several polyphenols, mainly visualized as fluorescent spots at 366 nm after derivatization with NPR (Figure S1). HPTLC analysis was also performed on the extract from the pulp and the edible part of DR2 aubergine berries at both ripening stages. The pulp represented the mesocarp of the fruit, obtained after peel separation, while the edible part was the pericarp after removing the calix. Also, control aubergine berries from a 67/3 variety of *S. melongena* obtained from the same experimental fields, was included in the analysis (Figure S1). The edible part exhibited a polyphenolic fingerprint similar to that of the relative peel, with lower amounts as evidenced by the fluorescence intensity of the spots; conversely, in pulp samples, several compounds disappeared, thus suggesting that peel represents the mainly phenolic-enriched part of aubergine berries. Comparing the control eggplant and DR2, a similar trend was found at different ripening stages, with some differences in the upper part of chromatogram (Figure S1). The HPTLC chromatograms highlighted the presence of rutin, chlorogenic acid, caffeic acid, and epicatechin in both DR2B and DR2C samples (Figure S2). Conversely, apigenin was a peculiar compound of DR2C extract (Figure S2, Table 2).

Among phytochemicals identified and determined by densitometric analysis, chlorogenic acid and rutin were found to be ubiquitous, with a significantly increased content in DR2B extract respect to DR2C (Figure S2; Table 2). The control 67/3C eggplant sample contained chlorogenic acid, rutin, apigenin, while it was lacking epicatechin and caffeic acid (Figure S1). Regarding anthocyanins, D3R was found to be the most abundant compound at both ripening stages, with low amounts of NAS and delphinidin-3-*O*-β-d-glucoside: the ratio between D3R and NAS was about 18 and 19 for DR2B and DR2C, respectively; this confirms that it represents the characteristic anthocyanin of DR2 eggplant variety, in agreement with what reported by Mennella et al. [33].

Anthocyanins were found abundant in these extracts from aubergine, with a total amount of 0.53 ± 0.012 mg cyanidin-3-glucoside equivalent and 76.44 ± 3.82 mg of D3R equivalent per 100 g of fresh fruit [39,41]. The acidified ethanolic extract from eggplant peel was found to contain 62.92 ± 0.15 mg delphinidin-3-glucoside equivalent/100 g of extract, with a ratio between anthocyanins and flavonoids of about 4 [42].

Although for our samples the total anthocyanin amount was not determined, taking into account that DR2 eggplant mainly produces delphinidin-3-glucoside as a peculiar anthocyanin [33], we estimated the ratio between anthocyanins and flavonoids was about 35 and 9 for DR2B and DR2C, respectively. This suggests that ripening increases the flavonoid amount, although anthocyanins were important constituents of eggplant peel. DR2B and DR2C extracts were also characterized by the presence of chlorogenic acid as a representative hydroxycinnamic acid, with a significant lowered amount (about 2.4-fold lower than DR2B) for ripening stage C. The seasonal variations of phenolic compounds have been also reported for other Solanaceae species: a decreased concentration of chlorogenic acid was found in long eggplants (about 70% lower levels), as well as in other species such as tomato (about a 43% reduced levels) with rising temperatures due to the summer season [43,48]. The levels of phenolic acids have also been found increased in relation to the organic growth, thus suggesting that they can be produced as a plant response to environmental stressors [49].

For eggplant varieties containing D3R, Mennella et al. [33] reported a 1.5-fold reduction of chlorogenic acid and total polyphenols ratio at the physiological ripening stage, with respect to the commercial one. For our samples, we found that the chlorogenic acid and total polyphenols ratio was about 1.5 and 0.4 for DR2B and DR2C extracts, respectively, with a reduction of 3.7-fold: this can be explained on the basis of the lower amounts of chlorogenic acid and the high anthocyanin levels in eggplant peel with respect to the pulp.

The phenolic fingerprint of the extracts was also detected by HPLC-PDA analysis [50], showing a different pattern for DR2B and DR2C samples (expressed as μg/mg of dry extract) (Table 3; Figure S3). Collectively, DR2B extract was characterized by a predominant presence of chlorogenic acid and a low amount of gallic acid, while DR2C displayed appreciable amounts of chlorogenic acid, *t*-ferulic acid, and *t*-cinnamic acid, along with small quantities of gallic acid, vanillic acid, naringin, syringic acid, 3-OH-4-MeO benzaldehyde, and 2,3-diMeO benzoic acid (Table 3).

García-Salas et al. [43] previously found the whole long eggplant to contain the flavonols quercetin 3,4′-*O*-diglucoside and kaempferol-3-*O*-rutinoside, hydroxycinnamic acids, homovanillic acid hexose, and delphinidin-3-*O*-rutinoside. Accordingly, Singh et al. [51] reported eggplant contains chlorogenic acids, with traces of quercetin and myricetin glucosides. Hydroxycinnamic acid conjugates were peculiar to the whole aubergine berry and pulp [44]; however, chlorogenic acid, ferulic acid, and caffeic acid were also found in the eggplant peel [38,52]. A complex flavonol profile was described for the eggplant skins [49], in spite of a low amount for the pulp [51]. Our results on the peel extracts from DR2 aubergine berries partly agree with literature evidence, mainly regarding the hydroxycinnamic acid composition, while the flavones apigenin and naringenin were firstly identified. Naringenin was also found to be transported and incorporated in the outer cuticle matrix [53], while apigenin was reported abundant in eggplant leaves [54].

### 2.2. DR2C Extract Exerts Cytoprotective Effect against Oxidative Cell Damage 

Considering the characteristic polyphenolic composition, DR2B and DR2C were assayed for their ability to interfere with the oxidative injury induced by the known oxidative agent *tert*-butyl hydroperoxide (tBOOH) in Caco2 cells, which represent a sensitive biological model to oxidant injury and have been widely used to study the effect of reactive oxygen metabolites and antioxidant agents [34]. The effect was evaluated under both normoxia and CoCl_2_-induced hypoxia, during which respiratory distress and oxidative stress occur as a result of the altered oxygen homeostasis [55]. In mammalian systems, CoCl_2_ is known to mimic hypoxia through stabilization of HIF-1α [56]. Preliminary experiments showed that DR2B-induced cytotoxicity signs starting from the concentration of 300 μg/mL, with a maximum inhibition of cell viability of about 30% with respect to control (Figure S4). Conversely, DR2C extract was nontoxic under both normoxic and hypoxic conditions, and slightly reduced the lactate levels extruded by treated cells (Figure S4). 

**Figure 1:**
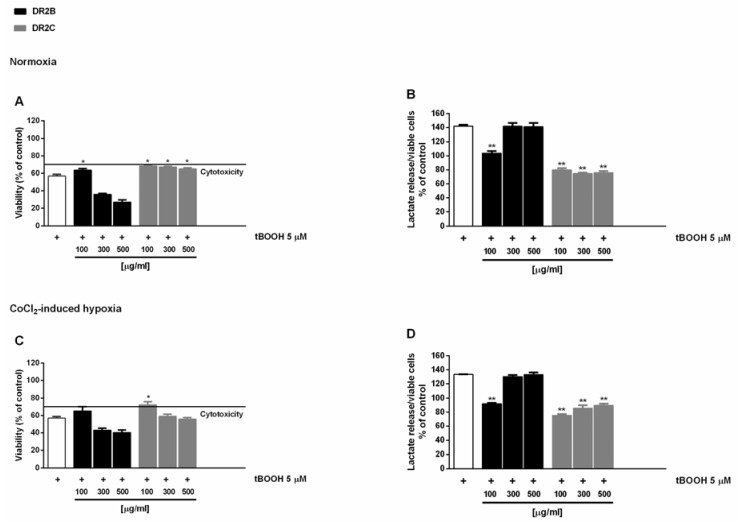
Effect of DR2B and DR2C extracts (100–500 µg/mL) on the oxidative damage induced by *tert*-butyl hydroperoxide (tBOOH; 5 μM) in Caco2 cells under normoxia and CoCl_2_-induced hypoxia. The pro-oxidant agent tBOOH was added to cells after a 24 h pre-treatment with the extracts. (**A**,**C**) Cell viability expressed as % of control. (**B**,**D**) Lactate levels normalized to the number of viable cells. Data are mean ± SE from two independent biological replicates, each one performed in two technical replicates (*n* = 2). * *p* < 0.05 and ** *p* < 0.01 vs. tBOOH by ANOVA followed by Dunnett’s multiple comparison post-test.

In the antioxidative cytoprotective assay, the pro-oxidant agent tBOOH reduced cell viability by about 40% with respect to the control under both normoxia and hypoxia (Figure 1A,C), with an increased lactate metabolism with respect to control of about 40% and 30%, respectively (Figure 1B,D). 

The DR2C extract displayed cytoprotective effects by inhibiting the tBOOH-induced cytotoxicity of about 21% and 23% under normoxia and hypoxia (Figure 1A,C): under these experimental conditions, lactate levels induced by tBOOH were found to be markedly reduced by DR2C (Figure 1B,D), reaching a maximum inhibition of 67% and 58% under normoxia and hypoxia. 

A slight but significant cytoprotection (lower than 10% increase of cell viability), with a 42% reduction of tBOOH-induced lactate metabolism, was also found at the lowest concentration of DR2B under normoxic conditions (Figure 1A,B). These results are remarkable, and highlight that the tested extracts can counteract the oxidative damage, thus blocking the tBOOH-induced upregulation of Warburg effect.

Caco2 cells, along with several types of cancer cells, are characterized by peculiar energetic requirements, due to their need to grow quickly with respect to normal cells. Therefore, they give a modified metabolism with increased aerobic glycolysis and lactate production. This glycolytic phenotype, known as the Warburg effect, leads to an increased nontoxic oxidative stress with a loss of antioxidant capabilities and seems to be responsible for a highly resistant and malignant behavior of cancer cells [57]. When Caco2 and other glycolytic cancer cells are subjected to an exogenous oxidative stressor, ROS increase over tolerable levels; therefore, resistant cells markedly upregulate their glycolytic metabolism to overcome oxidative injury [58]: as a consequence, high lactate levels are extruded by cells with an increased steady-state ROS condition [57]. In this context, affecting the glycolytic phenotype of Caco2 cells can represent a measure of the antioxidant power of the tested samples.

Literature evidence suggests a possible involvement of anthocyanins in the registered effects, since both NAS and D3R displayed protective properties in osteoclasts against the tBOOH-induced oxidative damage, likely by scavenging free radicals. This effect could be ascribed to the –OH moieties on the B ring, to the number of OH– moieties in total, or to the type and extent of glycosylation and acylation [59,60]. Along with anthocyanins, the tested samples are important source of phenylpropanoids, mainly chlorogenic acid (Table 3).

**Figure 2:**
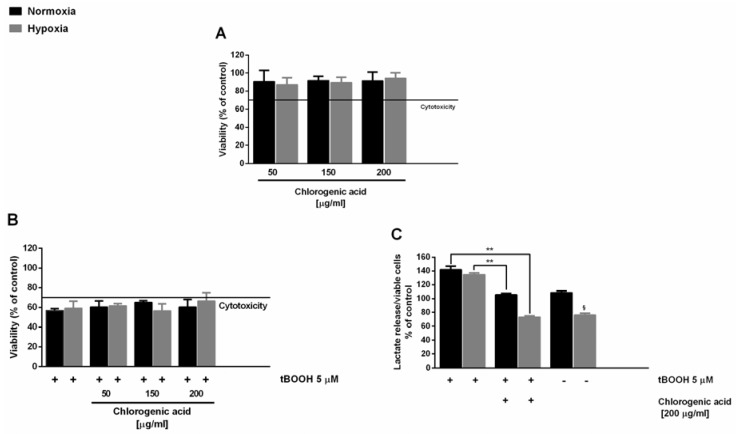
Effect of chlorogenic acid (50–200 µg/mL) on the oxidative damage induced by *tert*-butyl hydroperoxide (tBOOH; 5 μM) in Caco2 cells under normoxia and CoCl_2_-induced hypoxia. The pro-oxidant agent tBOOH was added to cells after a 24 h pre-treatment with the substance. (**A**) Cell viability was expressed as % of control. (**B**) Cell viability in the presence of the oxidative agent tBOOH expressed as % of control. (**C**) Lactate levels normalized to the number of viable cells. Data are mean ± SE from two independent biological replicates, each one performed in two technical replicates (*n* = 2). * *p* < 0.05 and ** *p* < 0.01 vs. tBOOH by ANOVA followed by Dunnett’s multiple comparison post-test.

In this context, here, we also evaluated the antioxidant cytoprotective activity of chlorogenic acid, in order to highlight its involvement in the activity of the extracts. Under our experimental conditions, chlorogenic acid induced nontoxic effects up to the highest concentration revealed in the DR2C extract (about 200 μg/mL), with a significant reduction of lactate levels under hypoxia (Figure 2A). When assessed for cytoprotection, the substance induced only a slight inhibition of the tBOOH-induced cytotoxicity (Figure 2B), in spite of a remarkable lowering of t-BOOH-induced lactate secretion (about 40% and 60% reduction under normoxia and hypoxia, respectively) (Figure 2C). This suggests that this compound can partly contribute to the activity of the extracts, although it does not represent the only bioactive constituent.

**Figure 3:**
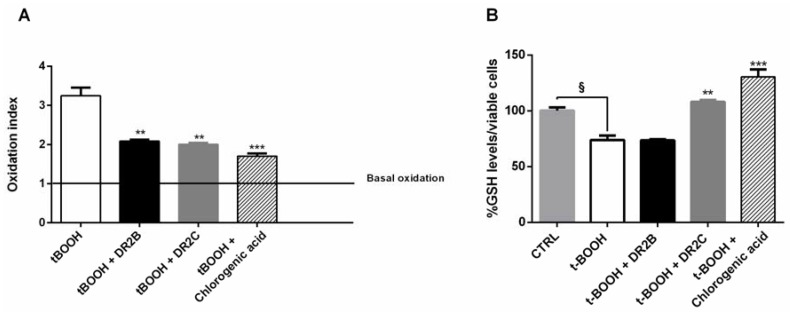
Effect of DR2B (100 µg/mL), DR2C (100 µg/mL), and chlorogenic acid (50 µg/mL) on the ROS and GSH levels induced by *tert*-butyl hydroperoxide (tBOOH; 5 μM) in Caco2 cells under normoxia. (**A**) Reactive oxygen species (ROS) levels expressed as oxidation index with respect to the basal levels. (**B**) GSH levels measured in cell lysates were normalized to viable cells and expressed as a percentage (level from control cells was considered 100%). Data are mean ± SE from at least two independent biological replicates (*n* = 2). ^§^ *p* < 0.01 vs. control; *** *p* < 0.001 and ** *p* < 0.01 vs. tBOOH by ANOVA followed by Dunnett’s multiple comparison post-test.

In order to better characterize the antioxidant cytoprotective effects of the tested samples, we also measured if the treatments may affect the intracellular content of ROS and GSH. In spite of a significant increase of ROS level induced by tBOOH (about 3-fold increase), both DR2B and DR2C extracts, and chlorogenic acid, significantly lowered the tBOOH-induced ROS levels, with a reduction of about 32%, 35%, and 45% respectively (Figure 3A). 

GSH levels were found reduced by tBOOH by about 25% with respect to the control; DR2B extract did not affect the tBOOH-induced GSH levels, while in the presence of DR2C and chlorogenic acid, the GSH content was significantly restored with respect to tBOOH treatment (about 1.5- and 1.8-fold, respectively) (Figure 3B). These results suggest that DR2C extract and chlorogenic acid not only counteract the oxidative damage of tBOOH, but also restore the antioxidative cell defenses, which are known to be downregulated in cancer cells, and modulate lactate production: altogether, these mechanisms can contribute to their antioxidative cytoprotective effects. However, the involvement of other specific factors or signaling pathways cannot be excluded. 

The present results agree with a previous study, in which eggplant stalks were found to possess cytoprotective, antioxidative, and anti-inflammatory properties, thus suggesting possible further interest for eggplant product waste as a source of nutraceuticals and new pharmaceutical agents [61].

### 2.3. Antioxidant Activity Assays

Different antioxidant mechanisms, including radical scavenging and chelating activity, were also evaluated for the DR2B and DR2C extracts by spectrophotometric assays. The scavenging activity was evaluated against both synthetic DPPH^•^ and ABTS^•+^ radicals and against the hydroxyl radical.

**Figure 4:**
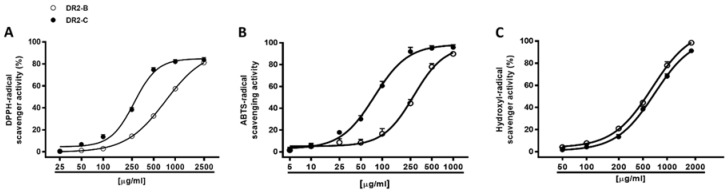
Scavenging activity of DR2B and DR2C extracts against DPPH (**A**), ABTS (**B**), and hydroxyl (**C**) radicals. Data are mean ± SE from two independent experiments, with each one performed with 3 technical replicates (*n* = 6).

Under our experimental conditions, both DR2B and DR2C extracts (5–2500 μg/mL) inhibited, in a significant and concentration dependent manner, the DPPH^•^ and ABTS^•+^ radicals, with a higher potency of DR2C (Figure 4A,B): the IC_50_ value of DR2C was about 2.7- and 3.6-fold lower than that of DR2B (Table 4). The positive control Trolox was about 54- and 39-fold more effective than DR2C against DPPH and ABTS, respectively.

According to the Pearson analysis, the scavenger activities against DPPH and ABTS radicals were significantly correlated for both DR2B and DR2C extracts (Table 5 and Table 6), although they exhibited higher potency against ABTS with respect to DPPH radical (2.6- and 3.4-fold respectively). DPPH is a pre-existing radical which can be neutralized by electron or hydrogen transfer, thus forming a stable diamagnetic molecule; conversely, ABTS cation, generated just before the experiments through different activators, requires an electron-transfer process to be scavenged, and is more reactive than DPPH [62]. These important chemical features can lead to different affinities and kinetics reactions, and in the scavenging potencies. Our results suggest a major involvement of electronic transfer as scavenging mechanism. Taking into account ABTS requires an aqueous reaction media; in spite of the methanolic or ethanolic environment of DPPH, we hypothesize that radical scavenging effects of DR2B and DR2C extracts are mainly due to polar bioactive constituents.

The extracts were also assayed for their ability to neutralize hydroxyl radical, thus resulting in both being able to scavenge this ROS metabolite, in a significant and concentration-dependent manner, with DR2B being slightly more potent than DR2C (Figure 4C; Table 4). The positive control Trolox resulted at least 10-fold more potency than the tested extracts (Table 4).

As estimated by the Pearson analysis, for all the samples, DPPH, ABTS, and hydroxyl radical scavenger activities were significantly correlated, with a correlation coefficient from 0.91 to 0.99 (Table 5 and Table 6). The correlation of DPPH and hydroxyl radical scavenger activity could be due to their common high reactivity, and suggests the presence in the extracts of some constituents able to counteract both species, among which includes phenolic compounds with multiple hydroxyl groups (i.e., caffeic acid and gallic acid) [63]. However, due to the low levels of these compounds in the extracts (Table 3), high concentrations of the samples are required for exerting the scavenger activity. Taking into account the phytochemical composition (Table 2 and Table 3), the contribution of phenolic acids, flavonoids (or flavonoid glycosides), and anthocyanins to the radical scavenger activity of the extracts is expected. In fact, numerous naturally occurring flavonoids have been highlighted to possess hydroxyl radical scavenging properties, and to interfere with other ROS species, thus suggesting their important protective role for health [64].

**Figure 5:**
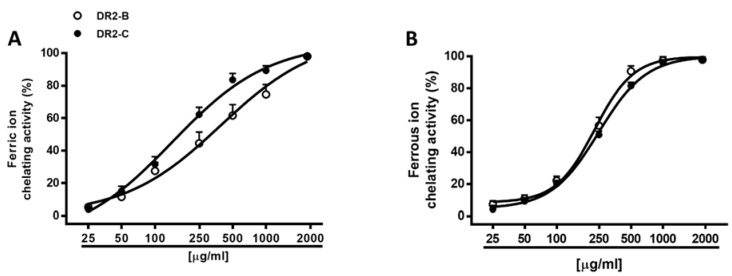
Chelating activity of DR2B and DR2C extracts against ferric (**A**) and ferrous (**B**) ions. Data are mean ± SE from two independent experiments, each one performed in 3 technical replicates (*n* = 6).

Hydroxyl radical is a strongly reactive radical which can cause serious damage to biomolecules, such as lipids, proteins, and nucleic acids. Due to its extremely high reaction rate, reducing its generation, for instance, by blocking the Fenton reaction, appears to be a suitable antioxidant strategy for preventing its damage. ROS species production can be facilitated by elemental species, such as iron: ferrous ions induce metal-catalyzed oxidation and participate in hydroxyl radical-generating Fenton type reactions; ferric ions also produce radicals from peroxides, although the rate is 10-fold less than that of ferrous ion [65]. Here, we assessed the ability of DR2B and DR2C extracts to indirectly affect ROS generation by reducing and/or chelating iron through the formation of ferrous/ferrozine complex [66]. Under our experimental conditions, both samples exerted a weak ferric reducing activity, thus hindering evaluation of IC_50_ (Table 4). Conversely, they were effective as chelators of both ferrous and ferric ions, with almost 3-fold high potency of DR2C as chelators of ferric ions (Figure 5A), in spite of a similar potency against ferrous ions (Figure 5B). As estimated by the Pearson analysis, the chelating and radical scavenging activities of the extracts appeared to be significantly correlated among them (Table 5 and Table 6).

The antioxidant capacity of eggplant is ranked in the top ten of 120 different vegetables, although some differences can occur due to variety, fruit shape, and size and methodology [25]. Jung et al. [40] and Nisha et al. [41] reported the DPPH radical scavenging and metal chelating properties of eggplant peel, while Kaneyuki et al. [67] found strong scavenging effects against hydroxyl radical generation. Different extracts from fresh aubergine berry peel produced radical scavenging effects against DPPH and hydrogen peroxide, and metal chelation, which were mainly related to phenolics [42]. Singh et al. [49] also described the ability of skin eggplant to inhibit the cupric ion-mediated lipoperoxidation. Likewise, aubergine berries exhibited radical scavenging and chelating activities [26,68]. Mennella et al. [33] also suggest the involvement of several bioactive constituents to the scavenging activity of NAS-type (containing nasunin as major anthocyanin) and D3R-type (containing delphinidin-3-rutinoside as major anthocyanin) eggplant varieties. Higher antioxidant capacities were also reported for semi-purified peel extracts from D3R-type and NAS-type aubergine berries respect to the purified D3R and NAS anthocyanins [69], thus confirming that other phytochemicals besides anthocyanins are involved in the antioxidant activity of the phytocomplex. Accordingly, our results suggest that several components of the DR2B and DR2C phytocomplex can contribute to the described antioxidant activity, acting by direct (by neutralizing DPPH, ABTS, and hydroxyl radical) and/or indirect (as chelators of ferrous and ferric ions) mechanisms. Among phytochemicals, the antioxidant properties of chlorogenic acid and the esters formed between caffeic and quinic acids are widely reported [70]. On the other hand, the radical scavenging and chelating effects of different polyphenols and anthocyanins have been published [28,29,37,71]. Particularly, delphinidin glucosides have shown to possess the strongest scavenging activity against superoxide anion and peroxynitrite, and NAS has been demonstrated to be a potent superoxide anion radical scavenger [69]. However, along with polyphenols and anthocyanins, a possible role of other unidentified phytochemicals in the biological activity of DR2 eggplant peel cannot be excluded.

### 2.4. DR2C Extract Affects HSV-1 Replication

**Figure 6:**
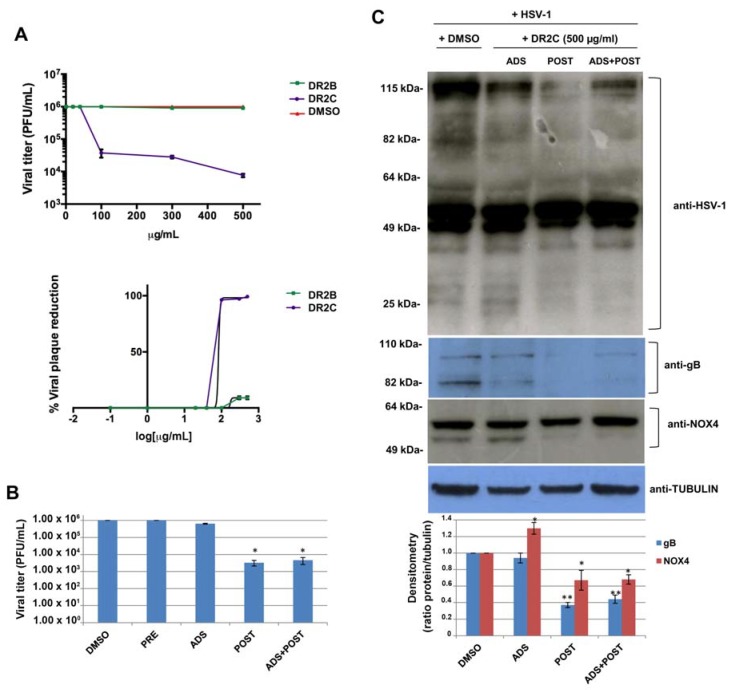
DR2C treatment affects HSV-1 replication. (**A**) Plaque reduction assay: viral titer of HSV-1-infected cells treated with increasing concentrations of DR2B and DR2C. Values are expressed as means ± SD of four experiments, each performed in duplicate. The IC_50_ of DR2C was calculated by regression analysis of the dose–response curves. (**B**) Time-of-addition assay: DR2C was added to Vero cells before (PRE), during (ADS), or after adsorption for the following 24 h (POST) or as double doses (ADS+POST). Supernatants were subjected to a standard plaque assay to evaluate viral titer. Data are expressed as means ± the SD of three independent experiments, each performed in duplicate (* *p <* 0.05 versus DMSO-treated HSV-1-infected sample). Cell lysates were analyzed by Western blotting (**C**) with anti-HSV-1, anti-gB, anti-NOX4, and anti-tubulin antibodies. Densitometric analysis of gB and NOX4 (lower band) is shown in the graph under the representative Western blot (* *p <* 0.05 and ** *p* < 0.01 vs. DMSO-treated HSV-1-infected cells).

Several studies have reported that viral infections are often associated with an imbalance in the intracellular redox state of host cell, that shifts towards pro-oxidant conditions [72,73]. Redox alterations are useful for the virus, since many pathways involved in the regulation of viral replication and host responses are highly responsive to even transient changes in the redox state of the cytoplasmic environment [74,75]. For this reason, several antioxidant molecules, both natural and synthesized, have been reported to exert antiviral activity [5,13,17,76,77,78]. Therefore, based on the above results relating to antioxidant properties of DR2B and DR2C extracts, their potential antiviral activity against HSV-1 was studied. To this aim, a plaque reduction assay was performed: confluent monolayers of VERO cells were infected with HSV-1 at multiplicity of infection (m.o.i.) of 1 for 24 h in the presence of increasing concentrations of both the extracts, ranging from 1 μg/mL to 500 μg/mL. DMSO-treated HSV-1-infected cells were used as control. Plaque formation was scored two days later. The dose–response curves, shown in Figure 6A, demonstrate that DR2C inhibited HSV-1 plaque formation in a dose-dependent manner with respect to DMSO-treated cells. In particular, the higher inhibition of viral replication was reached with 500 μg/mL of the extract (about 2.5 log inhibition compared with untreated infected cells). On the contrary, HSV-1 replication was not affected by DR2B treatment. Next, to demonstrate that the antiviral effect of DR2C was not a consequence of cytotoxicity, VERO cells were incubated with different concentrations (ranging from 1 to 500 μg/mL) of DR2C, and cell viability was evaluated by the trypan blue dye exclusion assay. As control, VERO cells were treated with DMSO. After 24 h of incubation, no significant alteration of cellular morphology and viability was detected by light microscope observations up to the concentrations of 500 μg/mL. Cell count of DR2C treated cells was compared to DMSO-treated and untreated cells, and no significant differences were observed (data not shown). Although the 50% inhibitory concentration (IC_50_) value for DR2C was 83.4 μg/mL (Figure 6A), the following experiments were performed using the extract at 500 μg/mL, as the highest antiviral activity was achieved with that dose, which was not cytotoxic.

At first, time-of-addition assays were performed. Vero cells were pre-incubated (PRE) with the extract for 3 h at 37 °C before the viral challenge (i.e., before virus adsorption phase), in order to analyze its possible interference with some cellular receptors and co-receptors used by HSV-1 to bind the host cell. Next, DR2C was added to VERO cells during the HSV-1 adsorption phase (ADS) (i.e., during the very early phase of virus lifecycle). Finally, the extract was administrated to the cellular monolayer after the viral adsorption phase (POST) for 24 h (i.e., during the early and late phases of the virus lifecycle). Furthermore, DR2C was also tested as a double dose during, and after, the HSV-1 adsorption period (ADS+POST). DMSO alone was administered as control. In all the experimental conditions, 24 h (post infection) p.i., supernatants of infected cells were collected and used to determine viral titer by standard plaque assay. As shown in Figure 6B, no difference in HSV-1 replication was observed in DR2C pre-treated cells compared to control (about 10^6^ pfu/mL in both cases). Similarly, DR2C treatment during viral adsorption did not significantly impair HSV-1 replication (6.5 × 10^5^ pfu/ml vs. 10^6^ pfu/mL). On the contrary, DR2C maintained in cellular culture medium for 24 h after viral adsorption significantly inhibited viral replication, confirming the trend observed in the previous plaque reduction assays (3.3 × 10^3^ pfu/mL vs. 10^6^ pfu/mL, i.e., about 2.6-log inhibition). Interestingly, the same HSV-1 titer reduction was observed in samples treated with a double dose of DR2C, confirming that the extract was not effective during the virus challenge and showing that the extract affected only the post adsorption phase of the virus lifecycle, including the synthesis of viral DNA and proteins. Thus, to check whether DR2C treatment altered viral protein synthesis, we performed a Western blot by using an antibody directed against the main HSV-1 proteins (ICPs and late). As shown in Figure 6C, DR2C administration to cells for 24 h after viral adsorption was able to reduce the expression of many viral proteins, particularly proteins with high and low molecular weight (over 80 kDa and lower 40 kDa, respectively). Furthermore, in the same experimental condition, the viral glycoprotein B (gB, late protein) expression was also strongly reduced, as confirmed by densitometric analysis (about 60% reduction compared to DMSO-treated infected cells) (Figure 6C).

The results obtained in HSV-1-infected cells treated with DR2C are in agreement with previous studies showing that several stages of HSV-1 replication, favored by oxidative conditions, are affected by modulation of intracellular redox state [17,79]. To note that although DR2B was able to partially reduce ROS production induced by tBOOH, it did not restore the GSH levels (see Figure 3B). Since reducing conditions in infected host cells are important for the control of viral replication, the inability of DR2B in restoring the imbalance in the redox state could, in part, explain its inefficacy against HSV-1 replication. On the contrary, according to the Pearson analysis, the anti-HSV1 activity of DR2C appears to be significantly correlated with both the radical scavenging and chelating activities (Table 7), thus suggesting that the samples can counteract the redox imbalance induced by virus in host cell.

One of the main intracellular sources of ROS is represented by the NADPH (nicotinamide adenine dinucleotide phosphate) oxidase (NOX) family, constituted by seven enzymes generating superoxide anion (O_2_^−^) or hydrogen peroxide (H_2_O_2_), from molecular oxygen [80], that regulate specific cellular processes. However, they are also activated during acute or chronic viral infections, i.e., influenza virus and HCV, playing an important role in the regulation of immune response to infections (isoform NOX2) [81,82] as well as viral replication (isoform NOX4) [75,83,84,85]. Therefore, we evaluated the effect of the DR2C on the expression level of NOX4 during HSV-1 infection. To this aim, HSV-1-infected VERO cells, treated with DR2C at different time of infection, were analyzed by Western blotting to evaluate NOX-4 expression. As shown in Figure 6C, the anti-NOX4 antibody recognized two NOX4 isoforms, and the one with a lower molecular weight was completely reduced by DR2C treatment after viral challenge (POST). It is known that NOX4 is highly expressed in the kidney [86], and that in the human, there are many NOX4 isoforms produced as splice-variants [87], some of which may have different functions in ROS-related cell signaling. Unfortunately, scientific literature does not report evidence about NOX4 isoforms in *Cercopithecus aethiops* (from which Vero cells are derived); however, we would mention as evidence that 227 organisms have orthologs with human gene *NOX4* (as reported in gene bank HomoloGene: *41065*). Further studies are needed to clarify this aspect. 

Among the phytoconstituents of DR2C extracts, as highlighted at phytochemical analysis (Table 2 and Table 3), several phenylpropanoids (i.e., chlorogenic acid, caffeic acid, and vanillic acid) and flavonoids have been reported to possess antiviral properties [88,89,90,91]. Along with phenylpropanoids, D3R is one of the most represented compounds in the DR2C extract, as found by HPTLC densitometric analysis (Table 2). This compound has been reported to possess several antioxidant properties, and to be able to protect osteoblastic cells against oxidative damage induced by *tert*-butyl hydroperoxide [29,69]. Therefore, we tested whether the anti-HSV-1 activity of DR2C was mainly due to this compound. To this aim, D3R was added to VERO cells at concentration of 5 µg/mL (corresponding to 1% (*w*/*w*) of the first effective DR2C concentration against HSV-1) after HSV-1 adsorption for the following 24 h; cellular supernatant was collected to perform a standard plaque assay. The treatment with D3R did not inhibit HSV-1 replication (4 × 10^5^ PFU/mL treated vs. 3.8 × 10^5^ PFU/mL not-treated), suggesting that DR2C anti-HSV-1 activity depends on the whole phytochemical complex.

Altogether, our results indicate that DR2C is able to significantly impair HSV-1 propagation and viral protein expression in an in vitro model of VERO cells. To date, the mechanisms underlying the inhibition are not well defined, however, it is possible to speculate a control by DR2C of NOX4 activity and redox-regulated pathways during HSV-1 infection. 

Importantly, DR2C treatment could also affect viral replication by raising the intracellular GSH levels in HSV-1-infected VERO cells. In fact, we demonstrated, in the cytoprotective assay above-described, that DR2C is able to restore the GSH intracellular content in tBOOH-treated Caco2 cells (Figure 3B). It is known that different acute and chronic viral infections, including that of Herpes virus, induce a drop of GSH levels in infected cells, which favors viral propagation [17,18]. The administration of GSH or GSH derivative strongly inhibits virus replication by affecting specific steps of virus lifecycle and restores the GSH content in infected cells. However, further studies are in progress in our lab, aimed at clarifying this aspect. The modulation of redox state, through the re-establishment of reducing conditions in infected cells, may represent a good weapon to contrast viral infection with the advantage that the virus could have a lower probability to develop resistance.

## 3. Materials and Methods

### 3.1. Chemicals

All the chemicals, if not otherwise specified, and the RPMI 1640 medium were obtained from Sigma-Aldrich Co. (St. Louis, MO, USA). Sodium carbonate, Folin–Ciocalteu’s phenol reagent, tannic acid, aluminum chloride hexahydrate, and the analytical grade solvents ethyl acetate (AcOEt) and *n*-butanol, were purchased from Merck (Darmstadt, Germany). Standard flavonoids and phenylpropanoids (>95% purity) were obtained from synthesis, and checked by nuclear magnetic resonance spectroscopy. Delphinidin-3-rutinoside (D3R) and nasunin (NAS) were isolated and identified from the aubergine berry peel, according to previous methods [28,29].

Fetal bovine serum was obtained from Gibco, while the other reagents for antiviral studies, if not otherwise specified, were purchased from Invitrogen (Carlsbad, CA, USA). 

Anisaldehyde was prepared as a 0.5% (*v*/*v*) solution in an ice-cooled mixture of sulfuric acid, methanol, and acetic acid (1:17:2 *v*/*v*/*v*). Natural product reagent (NPR) was obtained by preparing a 0.5% (*w*/*v*) solution of diphenylborinic acid aminoethylester in AcOEt.

### 3.2. Plant Material and Extract Preparation

Fruits of *Solanum melongena* L. DR2 homozygous parental line were harvested from open-field grown plants at the experimental farm in Montanaso Lombardo, at two different fruit ripening stages, i.e., commercial (B, approximately 38 days after flowering) and physiological ripening (C, approximately 60 days after flowering). DR2 eggplant is the female parent of the F1 hybrid “Rimina”, and produces long-shaped dark purple fruit [92]. According to Mennella et al. [33], the fruits at stage B had reached their final size, and the skin color became less brilliant, with a green ring next to the skin and a less greenish pulp; the fruits harvested at stage C had an increased firmness, with a peel color that turned brownish and a spongy and white-yellowish pulp. 

The peel from DR2B and DR2C aubergine berries was carefully removed from the pulp, then the extracts were prepared by maceration in acidified (pH 3) 70% (*v*/*v*) EtOH (1:10 drug–solvent ratio or DSR) and room temperature. The extracts were filtered through no. 1 Whatman paper, and the solvent was evaporated under vacuum, thus obtaining the dried peel extracts with DER (drug–extract ratio) values of 1:23 and 1:25 for DR2B and DR2C aubergine berries, respectively. 

### 3.3. Phytochemical Analysis

#### 3.3.1. Determination of Total Polyphenol and Flavonoid Content

The total polyphenol and flavonoid contents were determined according to Di Sotto et al. [62]. The total polyphenol content was calculated as chlorogenic acid equivalents (CAE) per milligram of sample, while total flavonoids were expressed as quercetin equivalents (QE) per milligram of sample.

#### 3.3.2. High-Performance Thin-Layer Chromatography and Densitometric Analysis

High-performance thin-layer chromatography (HPTLC) and densitometric analysis were carried out according to Di Sotto et al. [66]. The peel extracts of *S. melongena* DR2B and DR2C (30 mg/mL in ethanol) were analyzed in comparison with known polyphenols (e.g., chlorogenic acid at concentrations of 0.25, 0.5, and 1 mg/mL, while caffeic acid, apigenin, epicatechin, and rutin, of 1 mg/mL). A HPTLC fingerprint was also determined for anthocyanins, by using NAS (or delphinidin-3-*p*-coumaroylrutinoside-5-glucoside), D3R, and delphinidin 3-*O*-β-d-glucoside (1 μg/mL) as standard compounds. Polyphenols and anthocyanins were identified by comparison with selected standards (Rf values, colors, UV spectra), then a densitometric analysis was performed by means of a CAMAG DigiStore2 digital system with winCATS software 1.4.3. Repeatability was determined by running a minimum of three analyses.

#### 3.3.3. HPLC-PDA Analysis

HPLC-PDA phenolic pattern was evaluated by a HPLC Waters liquid chromatography (model 600 solvent pump, 2996 PDA, Waters S.p.A., Milford, MA, USA), according to a previous standardized method [50]. The peel extracts of *S. melongena* DR2B and DR2C were weighed (1 mg/mL) and dissolved in mobile phase, and 20 µL was directly injected into the HPLC-PDA system. For over-range samples, 1:10 dilution factor was applied.

### 3.4. Cytoprotective Activity against Oxidative Stress 

#### 3.4.1. Cell Culture and Treatment

The human colorectal adenocarcinoma (Caco2) cells, obtained from ATCC (American Type Culture Collection, Manassas, VA, USA), were grown at 37 °C in 5% CO_2_ in Dulbecco’s modified Eagle’s medium, supplemented with sodium pyruvate (1%), fetal bovine serum (10% *v*/*v*), glutamine (2 mM), streptomycin (100 µg/mL), and penicillin (100 U/mL). Some experiments were also performed under hypoxia, obtained by culturing cells in a culture medium supplemented with the known chemical hypoxia inducer cobalt chloride (CoCl_2_; 100 μM), according to previously published methods [93]. All experiments were performed when cells reached the logarithmic growth phase.

#### 3.4.2. Cytotoxicity and Cytoprotection Assay

The cells were seeded into 96-well microplates (2 × 10^4^ cells/well) and allowed to grow for 24 h; then, progressive dilutions of the DR2B and DR2C extracts in dimethyl sulfoxide (DMSO; 100% (*v*/*v*)) were added to cells (1% (*v*/*v*) in the cell medium). The vehicle DMSO was nontoxic at final concentration of 1% (*v*/*v*) in the medium. Cytotoxicity was measured after 24 h incubation with the samples by the 3-(4,5-dimethylthiazol-2-yl)-2,5-diphenyl tetrazolium bromide (MTT) assay according to previous published methods [94]. The assay was carried out three times and, in each experiment, each concentration was tested almost in six technical triplicates. Cell viability was determined as follows:

[(OD treated cells − OD medium control)/(OD untreated cells − OD medium control)] × 100
(1)

In the cytoprotection assay, after a 24 h pre-treatment with DR2B and DR2C peel extracts, a low-toxic concentration (about 40% cytotoxicity as found in preliminary experiments) of the pro-oxidant agent tBOOH (5 μM) was added for 2 h to cells, then, the cell viability was measured as described above [79].

#### 3.4.3. Determination of Lactate Secretion under Normoxic and Hypoxic Conditions

The levels of lactate, released in the cell medium as a consequence of the glycolytic metabolism, was measured in both normoxic and hypoxic conditions by the spectrophotometric method described by Borshchevskaya et al. [95], with some changes. To perform the assay, the cells were subjected to treatment with DR2B and DR2C extracts (500 μg/mL) for 24 h, both alone and in the presence of tBOOH (5 μM; 2 h exposure), then, the cell medium was collected for lactate levels determination. To this end, the cell medium (50 μL) was added with a solution of iron(III) chloride (150 μL; 0.01% *w*/*v* in acetate buffer) and mixed for five minutes, then the absorbance of the formed iron(III) lactate was measured at 390 nm by a microplate reader (Epoch Microplate Spectrophotometer, BioTeK^®^ Instruments Inc., Winooski, VT, USA). The lactate levels were normalized to viable cells and expressed as percentage of the vehicle control.

#### 3.4.4. Intracellular Levels of Reactive Oxygen Species (ROS) Determination

The ROS levels induced by treatments were measured by the 2,7-dichlorofluorescein diacetate assay (DCFH-DA), according to Di Giacomo et al. [96] with slight changes. To perform the assay, 5 × 10^5^ cells were plated into 6-well culture wells. After 24 h incubation, confluent cells (about 60–70% of confluence) were treated with DR2B and DR2C extracts (500 μg/mL) for 24 h, then a low-toxic concentration (about 40% cytotoxicity as found in preliminary experiments) of the pro-oxidant agent tBOOH (5 μM) was added for 2 h to cells. Thereafter, the cells were added with DCFH-DA (10 µM; 6 μL), further incubated for 30 min, then collected and washed twice with HBSS (1×). Fluorescence was measured at an excitation wavelength of 485 nm and an emission wavelength of 528 nm, using a BD Accuri™ C6 flow cytometer (BD Biosciences, San Jose, CA, USA). In each experiment, a vehicle control (corresponding to the basal ROS level) and the pro-oxidant agent t-BOOH were also included; furthermore, the extracts alone were assayed to evaluate their effect on the basal ROS levels, released as a consequence of cell metabolism. The mean DCF fluorescence of 1 × 10^4^ cells was measured from all the treatments. The oxidation index was obtained by the ratio between the DCF fluorescence of the sample and that of the vehicle control.

#### 3.4.5. Intracellular Levels of Reduced Glutathione (GSH)

Intracellular levels of GSH were determined by the method of Ellman described by Vitalone et al. [97] with some changes. Briefly, after 24 h treatment, Caco2 cells were suspended in ice-cold hypotonic lysis buffer (50 mM Tris/HCl pH 7.5, 150 mM NaCl, 1 mM EDTA, 1% Triton X-100, 1 mM phenylmethylsulfonylfluoride, 10 μg/mL aprotinin, and 0.1 mM leupeptin). After 30 min of incubation at 4 °C, lysates were centrifuged at 14,000× *g* rpm for 30 min, and the supernatants were collected. Then, equal volumes of supernatants and 5-trichloroacetic acid (10 % *w*/*v*) were mixed and centrifuged at 10,000× *g* rpm for 5 min. After deproteinization, supernatant (50 µL), PBS (125 µL) and Ellman’s reagent (25 µL; 30 mM 5,5′-dithiobisnitro benzoic acid in 100 mL of 0.1% sodium citrate) were mixed, and the absorbance of the yellow product was read at 412 nm by a microplate reader (Epoch Microplate Spectrophotometer, BioTeK^®^ Instruments Inc., Winooski, VT, USA). The GSH levels were normalized to viable cells and expressed as percentage.

### 3.5. Antioxidant Activity Assays

The experiments were performed in 96-multiwell microplates away from direct light, and repeated at least twice, with three technical replicates. To perform the assays, progressive dilutions of DR2B and DR2C extracts in 100% (*v*/*v*) EtOH were used. In each test, negative or positive controls (Trolox, rutin, and quercetin) and control wells containing only the tested samples were included. The absorbance was measured by a microplate reader (Epoch Microplate Spectrophotometer, BioTeK^®^ Instruments Inc., Winooski, VT, USA). Some wells containing only the extracts were also included, in order to determine their possible absorbance.

DPPH, ABTS, and hydroxyl radical scavenging activities were determined according to Di Sotto et al. [66]. In order to evaluate the ability of the samples to interfere with ROS-generation through the block of Fenton reaction, the iron chelating and reducing activities were tested [62]. Chelation ability was evaluated against both ferrous and ferric ions, respectively, while reducing power against ferric ions.

### 3.6. Antiviral Assay

#### 3.6.1. Cell Culture 

African green monkey kidney (Vero) cells, obtained from ATCC (American Type Culture Collection, Manassas, VA, USA), were grown in RPMI 1640 medium (Sigma Aldrich, St. Louis, MO, USA) containing 10% heat-inactivated fetal bovine serum (FBS, Gibco), glutamine (0.3 mg/mL), penicillin (100 units/mL), and streptomycin (100 µg/mL), at 37 °C, in an atmosphere of 5% of CO_2_. The effect of the extracts (1–500 μg/mL) on Vero cell viability was tested in vitro by the trypan blue (0.02% final concentration) exclusion assay.

#### 3.6.2. Virus Production and Infection

For virus production, monolayers of VERO cells in 75 cm^2^ tissue culture flasks were infected with HSV-1 (strain F) at a multiplicity of infection (m.o.i.) of 0.01. After 48 h at 37 °C, infected cells were harvested with 3 freeze-and-thaw cycles, cellular debris was removed with low-speed centrifugation, and the virus titer was measured by a standard plaque assay, as described above. The titer of the virus preparation was 10^9^ plaque forming units (PFU)/mL. The virus was stored at −80 °C until use. For in vitro HSV-1 infection, confluent monolayers of Vero cells in 24-well plates were incubated with HSV-1 at a m.o.i. of 1 for 1 h at 37 °C, to allow virus adsorption to the host cells (adsorption phase). Then, the medium was replaced with fresh medium supplemented with 2% FBS, and the plates were maintained for 24 h at 37 °C in an atmosphere of 5% CO_2_ (post-infection phase).

#### 3.6.3. Determination of Viral Yields

The antiviral activity of DR2B and DR2C extracts was evaluated by plaque reduction and standard plaque assays as previously described in Marcocci et al. [12]. Briefly, to perform a plaque reduction assay, Vero cells grown in 24-well plates were infected with HSV-1 at 1 m.o.i. and, after 1 h at 37 °C (viral adsorption period), were washed with PBS and the medium replaced with RPMI containing 1% carboxymethylcellulose (CMC), 2% FBS, and different concentrations of DR2B and DR2C extracts (1–500 μg/mL) or DMSO as control. After 36/48 h at 37 °C plaque formation was scored, and the viral titer calculated as PFU/mL. To perform a standard plaque assay, confluent monolayer Vero cells were infected with different dilutions of supernatant of infected cells, and after 1 h at 37 °C (viral adsorption phase), the medium was replaced with RPMI containing 2% CMC and 2% FBS. After 48 h at 37 °C, monolayers were fixed with cold methanol for 20 min at −20 °C, and then stained with a 0.5% crystal violet solution. The plaques were counted, and the viral titer calculated as PFU/mL.

#### 3.6.4. Time-of-Addition Assay

DR2C extract was added at the final concentration of 500 μg/mL at different times of HSV-1 infection: (i) cells were pre-incubated for 3 h (pre-viral adsorption phase) with DR2C; (ii) DR2C was added only during or after viral adsorption phase; (iii) the extract was added during and post-adsorption phase. After 24 h of infection, the supernatants were collected and used to determine HSV-1 titer with standard plaques assay, as described before.

#### 3.6.5. Western Blot Analysis

Vero cells were washed with PBS, resuspended in cold lysis buffer (10 mM Tris-HCl, 150 mM NaCl, 1 mM phenylmethylsulfonyl fluoride, phosphatase inhibitor mixture (Sigma, St. Louis, MO, USA), and 1% Triton X-100, pH 7.4), and incubated for 30 min on ice. After centrifugation (10,000× *g* for 30 min at 4 °C) the supernatants were collected and assayed to determine their protein concentration (Bradford method, Bio-Rad, Hercules, CA, USA). Equivalent amounts of proteins were separated with SDS-PAGE, and blotted onto nitrocellulose membranes for Western blot analysis. The membranes were blocked with 10% nonfat dry milk in TBS, 1% Tween-20 for 1 h at room temperature, and incubated with primary antibodies: goat polyclonal anti-HSV (AbD Serotec, Oxford, UK), mouse monoclonal anti-glycoprotein B (Santacruz Biotechnology, Dallas, TX, USA), rabbit polyclonal anti-NOX4 (Santacruz Biotechnology, Dallas, TX, USA), or mouse monoclonal anti-tubulin (Sigma Aldrich, St. Louis, MO, USA) at a final concentration of 1 µg/mL. Secondary antibodies were horseradish peroxidase-conjugated (Jackson ImmunoResearch, West Grove, PA, USA). Blots were developed with the Pierce ECL Plus Western Blotting Substrate (Thermo Scientific, Waltham, MA, USA) and subjected to densitometric scanning.

### 3.7. Statistical Analysis

All values are expressed as mean ± SE or mean ± SD, as indicated. Statistical analysis was performed by GraphPad Prism™ software (GraphPad Software, Inc., San Diego, CA, USA). The one-way analysis of variance (one-way ANOVA), followed by Dunnett’s multiple comparison post-test, was used to analyze the difference between treatments. Moreover, unpaired data were analyzed with Student’s *t*-test. The concentration–response curves were constructed using the “Hill equation”: E = Emax/[1 + (10^LogEC50^/A)^HillSlope^], where E is the effect at a given concentration of agonist, Emax is the maximum activity, IC_50_ is the concentration that produces a 50% of the inhibitory response, A is the agonist concentration in molar, and HillSlope is the slope of the agonist curve. *P* values of less than 0.05 (*p* < 0.05) were considered statistically significant. The concentration of the extracts required to reduce virus yield by 50% (IC_50_) was calculated by regression analysis of the dose–response curves. 

## 4. Conclusions

Eggplants are known around the world as a vegetable crop of nutritional interest, due to their content of high amounts of vitamins, phenolics, and anthocyanins. However, they have been used in traditional medicine as a remedy for several ailments, including skin diseases and infections. Particularly, slices of eggplant stalks and berry peel were applied on the skin to treat frostbites, burns, and skin warts (known to be induced by human papillomavirus), although the scientific basis is lacking. Here, we described the antioxidative cytoprotective properties and the anti-herpetic activity of an ethanolic DR2C extract, obtained from the berry peel of a DR2 eggplant, which has been selected to contain high levels of delphinidin-3-rutinoside (D3R). Physiological ripeness has been highlighted to be the only suitable stage at which the antiviral activity was exerted. A lack of antiviral activity of D3R under our experimental conditions suggests that other phytoconstituents can be responsible for the anti-HSV-1 activity of DR2C. Among them, literature data support the possible role of chlorogenic acid, although no effects were found for the DR2B extract, whose chlorogenic acid amount was higher than that of DR2C. Therefore, the contribution of other phenolics or phytochemical groups, such as glycoalkaloids, is expected. However, taking into account the possible synergistic and/or antagonistic interactions that can occur under a phytocomplex, the true pharmacodynamic and/or pharmacokinetic contribution of DR2C phytoconstituents remains to be clarified. 

To date, our study is the first demonstration of the in vitro anti-HSV-1 activity of eggplant peel, due to multitargeted mechanisms, including the restoration of intracellular redox state that HSV-1 modulates to promote its replication in the host cells. Further studies are needed to deeply characterize the possible application of DR2C extract as an anti-HSV-1 remedy.

Altogether, these results provide preliminary scientific evidence on the traditional use of eggplant, and highlight a possible new interest for aubergine berry peel, which often represents a waste product, as a nutraceutical and pharmaceutical source for further development of anti-herpetic remedies.

## Figures and Tables

**Table 1:** Amounts of total polyphenols and flavonoids in DR2B and DR2C extracts (*n* = 6).

Compound	DR2B	DR2C
	µg/mg Sample (Mean ± SE)	
Total polyphenols (CAE)	39.0 ± 0.08	67.0 ± 0.04 **
Flavonoids (QE)	3.3 ± 0.01a	9.8 ± 0.01b **

CAE, chlorogenic acid; QE, quercetin equivalents. ^a^ 1.8 ± 0.01 μg CAE/mg extract. ^b^ 5.3 ± 0.01 μg CAE/mg extract. ** *p* < 0.01 denotes significant increased levels respect to DR2B by Student’s *t*-test.

**Table 2:** Levels of polyphenols and anthocyanins measured by the HPTLC densitometric analysis in DR2B and DR2C extracts (*n* = 3).

Compound	DR2B	DR2C
	µg/mg Sample (Mean ± SD)	
Apigenin	nd	15.7 ± 0.8 **
Caffeic acid	1.7 ± 0.1	2.2 ± 0.1 *
Chlorogenic acid	60.7 ± 3.2	24.5 ± 1.8 **
Delphinidin 3-*O*-β-d-glucoside	10.1 ± 0.7	nd **
Delphinidin-3-rutinoside	115.0 ± 0.2	90.3 ± 2.6 **
Epicatechin	8.7 ± 0.4	5.3 ± 0.3 **
Nasunin	6.3 ± 0.4	4.7 ± 0.6 *
Rutin	1.9 ± 0.1	1.1 ± 0.1 *

nd, not detected. * *p* < 0.05 and ** *p* < 0.01 denote significant differences respect to DR2B by Student’s *t*-test.

**Table 3:** Phenolic composition of DR2B and DR2C extracts by HPLC- PDA analysis.

Compound	DR2B	DR2C
	μg/mg of Sample (Mean ± SD)	
3-OH-4-MeO Benzaldehyde	nd	BLD
Benzoic acid	nd	nd
2,3-diMeO Benzoic acid	nd	BLD
3-OH Benzoic acid	nd	nd
p-OH Benzoic acid	nd	nd
Catechin	nd	nd
Chlorogenic acid	62.7 ± 6.2	28.5 ± 3.1 **
*t*-Cinnamic acid	nd	2.3 ± 0.2 **
*o*-Coumaric acid	nd	nd
*p*-Coumaric acid	nd	nd
*t*-Ferulic acid	nd	7.3 ± 0.6 **
Gallic acid	0.6 ± 0.06	0.5 ± 0.05
Harpagoside	nd	nd
Naringenin	nd	nd
Naringin	nd	0.4 ± 0.04 **
Quercetin	nd	nd
Rutin	nd	nd
Sinapinic acid	nd	nd
Syringic acid	nd	BLD
Vanillic acid	nd	0.9 ± 0.09 **

BLD, below limit of detection; nd, not detected. ** *p* < 0.01 denotes significant differences respect to DR2B by Student’s *t*-test.

**Table 4:** IC_50_ values of DR2B and DR2C extracts and the positive controls in the antioxidant activity assays.

	DR2B	DR2C	Positive Control
	IC_50_ (CL) μg/mL		
DPPH scavenging activity	731.1 (681.7–784.2)	269.1 (207.60–348.9)	4.9 ^a^ (4.4–5.6)
ABTS scavenging activity	277.6 (216.0–356.8)	77.6 (62.0–97.2)	1.9 ^a^ (1.8–2.0)
Hydroxyl radical inhibition	595.1 (480.7–736.9)	727.3 (566.0–934.7)	87.6 ^a^ (83.2–91.2)
Fe^2+^-chelating activity	228.4 (185.9–280.6)	249.3 (210.6–295.0)	431.6 ^b^ (318.2–543.5)
Fe^3+^-chelating activity	391.1 (204.6–747.8)	143.2 (90.9–225.6)	45.2 ^c^ (13.1–75.5)
Fe^3+^-reducing activity	-	-	0.4 ^a^ (0.3–0.5)

- not evaluable since the maximum inhibition at the highest tested concentration was lower than 80%. CL, confidence limits. ^a^ Trolox; ^b^ rutin; ^c^ quercetin.

**Table 5:** Pearson correlation coefficient among antioxidant activity assays for DR2B extract.

	Pearson r (CL; R Square)				
	DPPH Scavenger Activity	ABTS Scavenger Activity	Hydroxyl Radical Scavenger Activity	Fe^2+^-Chelating Activity	Fe^3+^-Chelating Activity
DPPH scavenger activity	1	-	-	-	-
ABTS scavenger activity	0.96 ** (0.67–0.99; 0.92)	1	-	-	-
Hydroxyl radical scavenger activity	0.99 *** (0.98–0.99; 0.99)	0.95 * (0.41–0.99; 0.90)	1	-	-
Fe^2+^-chelating activity	0.89 ** (0.42–0.98; 0.80)	0.99 *** (0.95–0.99; 0.99)	0.90 * (0.31–0.99; 0.8)	1	-
Fe^3+^-chelating activity	0.96 *** (0.75–0.99; 0.92)	0.98 *** (0.80–0.99; 0.95)	0.97 ** (0.74–0.99; 0.94)	0.96 ** (0.73–0.99; 0.92)	1

* *p* < 0.05, ** *p* < 0.01, and *** *p* < 0.001, statistically significant correlation (two-tailed *t*-test). CL, confidence limits.

**Table 6:** Pearson correlation coefficient among antioxidant activity assays for DR2C extract

	Pearson r (CL; R Square)				
	DPPH Scavenger Activity	ABTS Scavenger Activity	Hydroxyl Radical Scavenger Activity	Fe^2+^-Chelating Activity	Fe^3+^-Chelating Activity
DPPH scavenger activity	1	-	-	-	-
ABTS scavenger activity	0.88 * (0.26–0.99; 0.78)	1	-	-	-
Hydroxyl radical scavenger activity	0.91 * (0.37–0.99; 0.83)	0.99 ** (0.86–0.99; 0.98)	1	-	-
Fe^2+^-chelating activity	0.99 *** (0.98–0.99; 0.99)	0.91 * (0.35–0.99; 0.82)	0.92 * (0.4–0.99; 0.84)	1	-
Fe^3+^-chelating activity	0.98 *** (0.88–0.99; 0.97)	0.96 ** (0.70–0.99; 0.93)	0.89 * (0.27–0.99; 0.79)	0.99 *** (0.92–0.99; 0.98)	1

* *p* < 0.05, ** *p* < 0.01, and *** *p* < 0.001, statistically significant correlation (two-tailed *t*-test). CL, confidence limits.

**Table 7:** Pearson correlation coefficient among antioxidant activity, cytoprotection against oxidative stress and antiviral activity for DR2C extract.

Anti-HSV1 Activity vs.	Pearson r (CL; R Squared)
DPPH scavenger activity	0.97 * (0.17–0.99; 0.95)
ABTS scavenger activity	0.93 * (0.1–0.99; 0.87)
Hydroxyl radical scavenger activity	0.94 * (0.1–0.99; 0.89)
Fe^2+^-chelating activity	0.94 * (0.31–0.99; 0.88)
Fe^3+^-chelating activity	0.97 ** (0.6–0.99; 0.94)
Cytoprotection against tBOOH-induced oxidative stress	nsc

* *p* < 0.05, ** *p* < 0.01 and *** *p* < 0.001, statistically significant correlation (two-tailed *t*-test). CL, confidence limits. nsc, not significant correlation.

## Supplementary Materials

The following are available online, Figure S1: High-performance thin-layer chromatography (HPTLC) of the polyphenolic compounds of DR2B and DR2C extracts, Figure S2: HPTLC (300 nm) chromatograms and densitometric analysis (peak list, Rf values and abundance of revealed compounds) of DR2B and DR2C extracts, Figure S3: HPLC-PDA (278 nm) chromatograms of DR2B and DR2C extracts, Figure S4: Effect of DR2B and DR2C extracts the viability and lactate secretion of Caco2 cells under normoxia and cobalt chloride (CoCl2)-induced hypoxia.
