# Supplementary material for: A Polyphenol Rich Extract from Solanum melongena L. DR2 Peel Exhibits Antioxidant Properties and Anti-Herpes Simplex Virus Type 1 Activity In Vitro
Source: Molecules. 2018 Aug 17;23(8):2066. doi: 10.3390/molecules23082066 (PMC6222547; doi:10.3390/molecules23082066)

Supplementary materials

Article

A polyphenol rich extract from *Solanum melongena* L. DR2 peel exhibits antioxidant properties and anti-herpes simplex virus type 1 activity *in vitro*

Antonella Di Sotto [1,*], Silvia Di Giacomo [1], Donatella Amatore [2], Marcello Locatelli [3], Annabella Vitalone [1,*], Chiara Toniolo [4], Giuseppe Leonardo Rotino [5], Roberto Lo Scalzo [6], Anna Teresa Palamara [2], Maria Elena Marcocci [2,#], Lucia Nencioni [2,#,*]

[1] Department of Physiology and Pharmacology, Sapienza University, P.le Aldo Moro 5, 00185 Rome, Italy
[2] Department of Public Health and Infectious Diseases, Istituto Pasteur Italia-Fondazione Cenci-Bolognetti, Sapienza University, P.le Aldo Moro 5, 00185 Rome, Italy
[3] Department of Pharmacy, University "G. D'Annunzio" of Chieti-Pescara, Via dei Vestini 31, 66100, Chieti, Italy
[4] Department of Environmental Biology, Sapienza University, P.le Aldo Moro 5, 00185 Rome, Italy
[5] Research Centre for Genomics and Bioinformatics (CREA-GB), Via Paullese 28, 26836 Montanaso Lombardo, Lodi, Italy
[6] Research Centre for Engineering and Agro-Food Processing (CREA-IT), Via Venezian 26, 20133 Milan, Italy
#These authors equally contributed to the work

Correspondence: antonella.disotto@uniroma1.it; annabella.vitalone@uniroma1.it; lucia.nencioni@uniroma1.it

Figure S1. High-performance thin-layer chromatography (HPTLC) of the polyphenolic compounds of DR2B and DR2C extracts. (A) Visualization at 366 nm without derivatization. (B) Visualization at 366 nm after Natural Product Reagent (NPR) derivatization. (C) Visualization at 366 nm after anisaldehyde/NPR derivatization. (D) Visualization under white light after NPR and anisaldehyde derivatization. (E) Visualization at 255 nm. The chromatograms correspond to: control aubergine peel at physiological ripeness (track 1); DR2C peel (track 2); control aubergine peel at commercial ripeness (track 3); DR2B peel (track 4); control aubergine pulp at physiological ripeness (track 5); DR2C pulp (track 6); control aubergine pulp at commercial ripeness (track 7); DR2B pulp (track 8); control aubergine edible part at physiological ripeness (track 9); DR2C edible part (track 10); control aubergine edible part at commercial ripeness (track 11); DR2B edible part (track 12); chlorogenic acid 0.25 µg/ml (track 13); chlorogenic acid 0.5 µg/ml (track 14); chlorogenic acid 1 µg/ml (track 15); caffeic acid (track 16); apigenin (track 17); epicatechin (track 18); rutin (track 19).

A

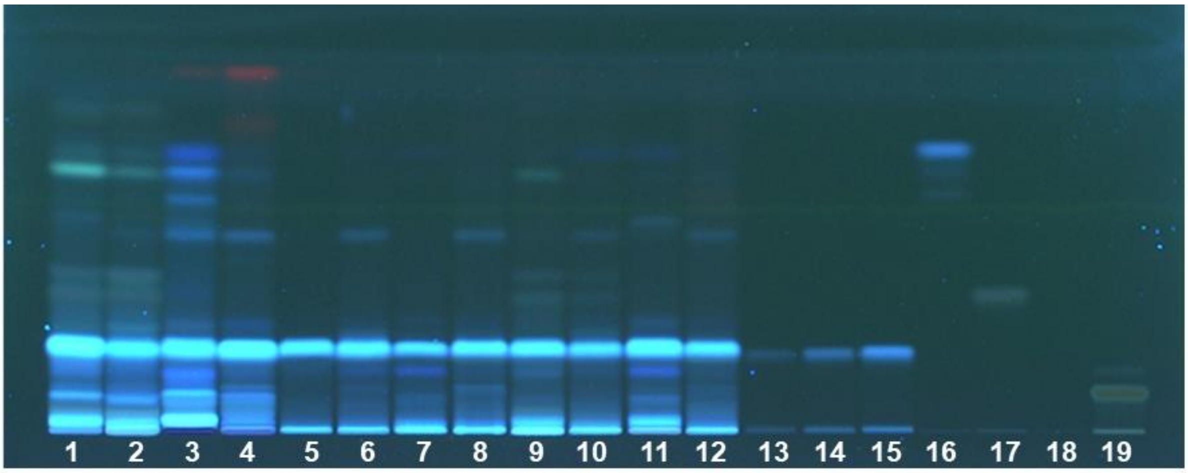

B

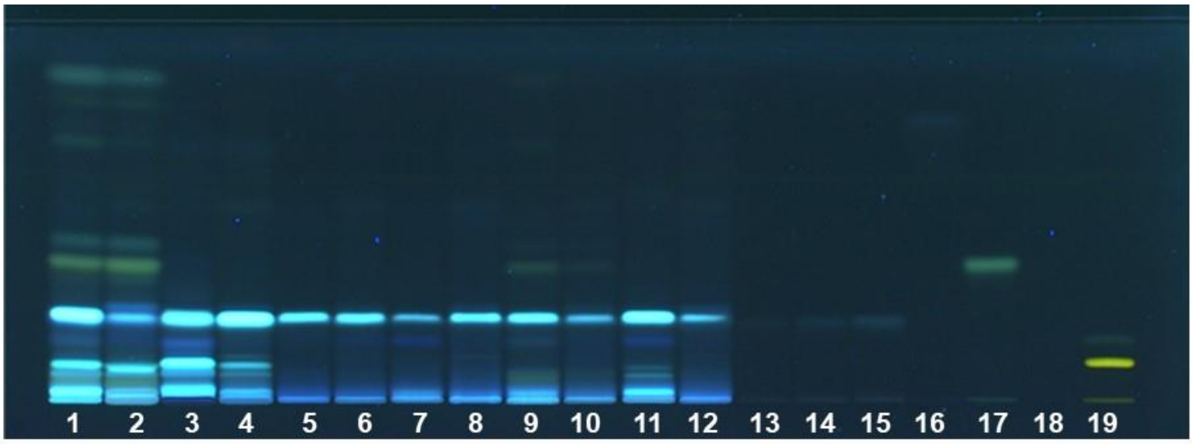

C

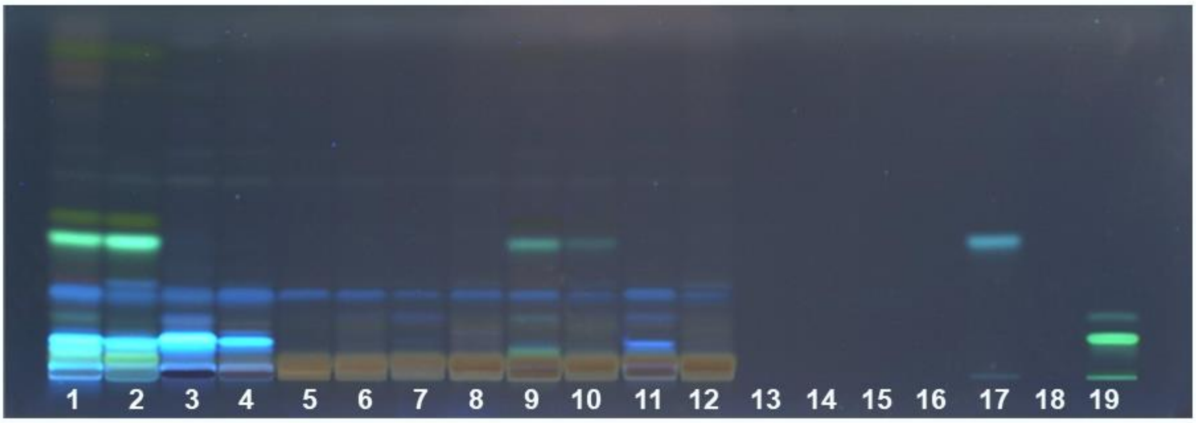
1 2 3 4 5 6 7 8 9 10 11 12 13 14 15 16 17 18 19

D

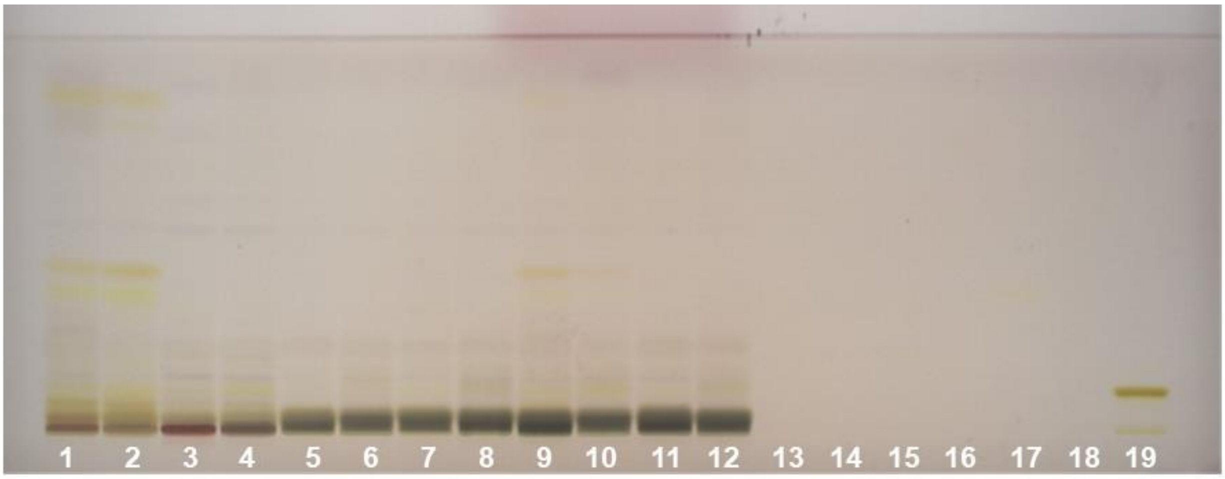
1 2 3 4 5 6 7 8 9 10 11 12 13 14 15 16 17 18 19

E

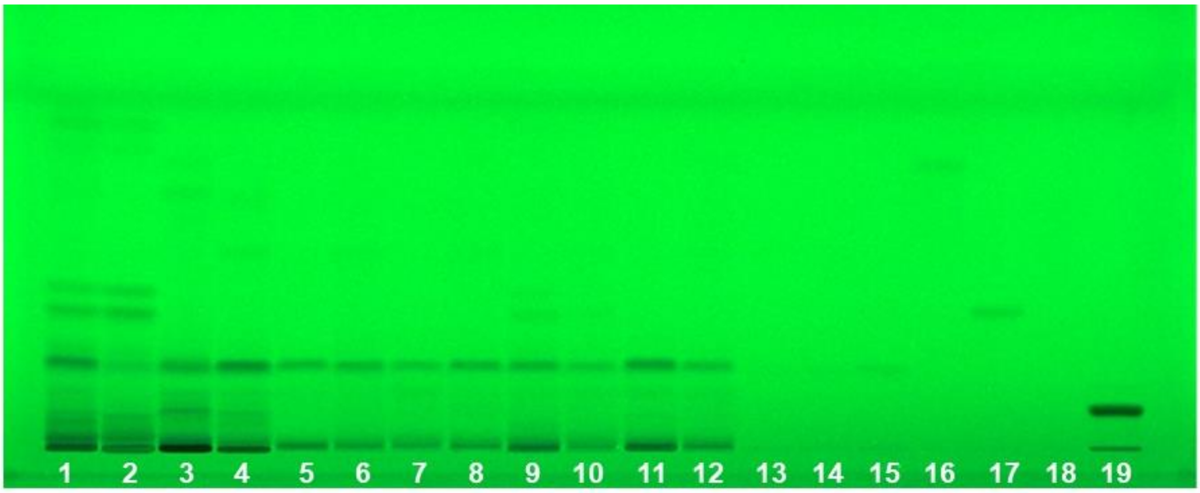
1 2 3 4 5 6 7 8 9 10 11 12 13 14 15 16 17 18 19

Figure S2. HPTLC (300 nm) chromatograms and densitometric analysis (peak list, Rf values and abundance of revealed compounds) of DR2B (A) and DR2C (B) extracts.

A

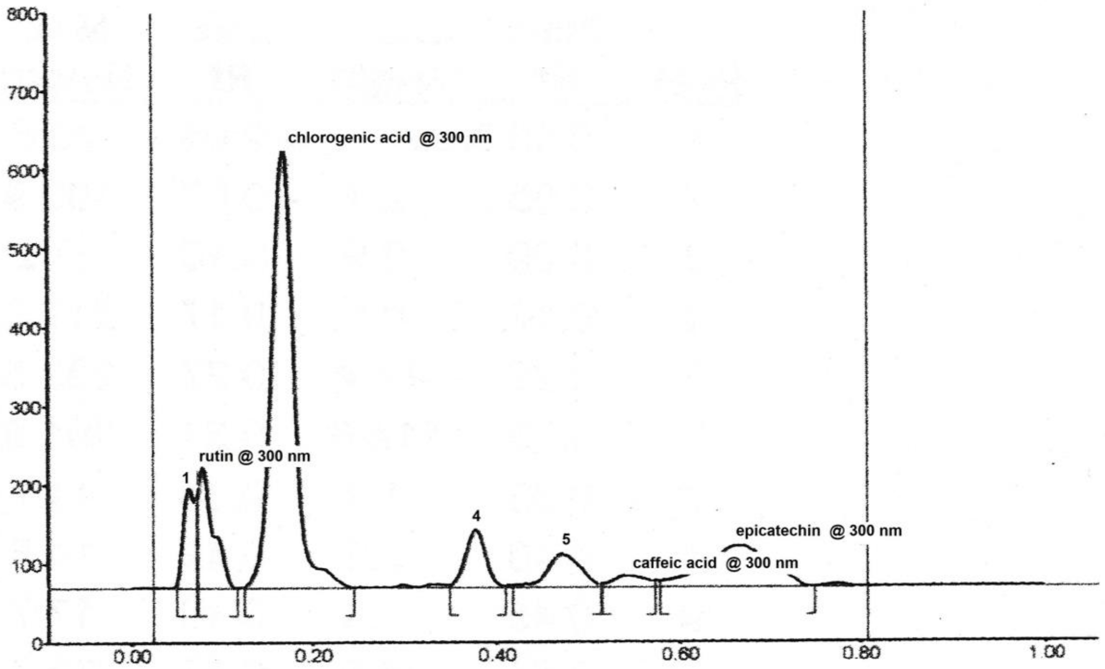

Peak	Start Rf	Start Height	Max Rf	Max Height	Max %	End Rf	End Height	Area	Area %	Assigned substance
1	0.05	0.3	0.06	126.9	12.47	0.07	108.0	1631.5	6.30	unknown
2	0.07	110.9	0.08	153.3	15.06	0.11	0.0	2670.1	10.31	rutin
3	0.12	0.1	0.17	557.0	54.74	0.24	0.1	14202.7	54.82	chlorogenic acid
4	0.35	2.9	0.38	72.8	7.16	0.41	1.8	1610.7	6.22	unknown
5	0.42	2.5	0.47	41.1	4.03	0.51	3.9	1524.8	5.89	unknown
6	0.51	3.9	0.54	14.4	1.41	0.57	7.6	513.3	1.98	caffeic acid
7	0.58	7.7	0.67	52.0	5.11	0.75	0.0	3755.2	14.49	epicatechin

B

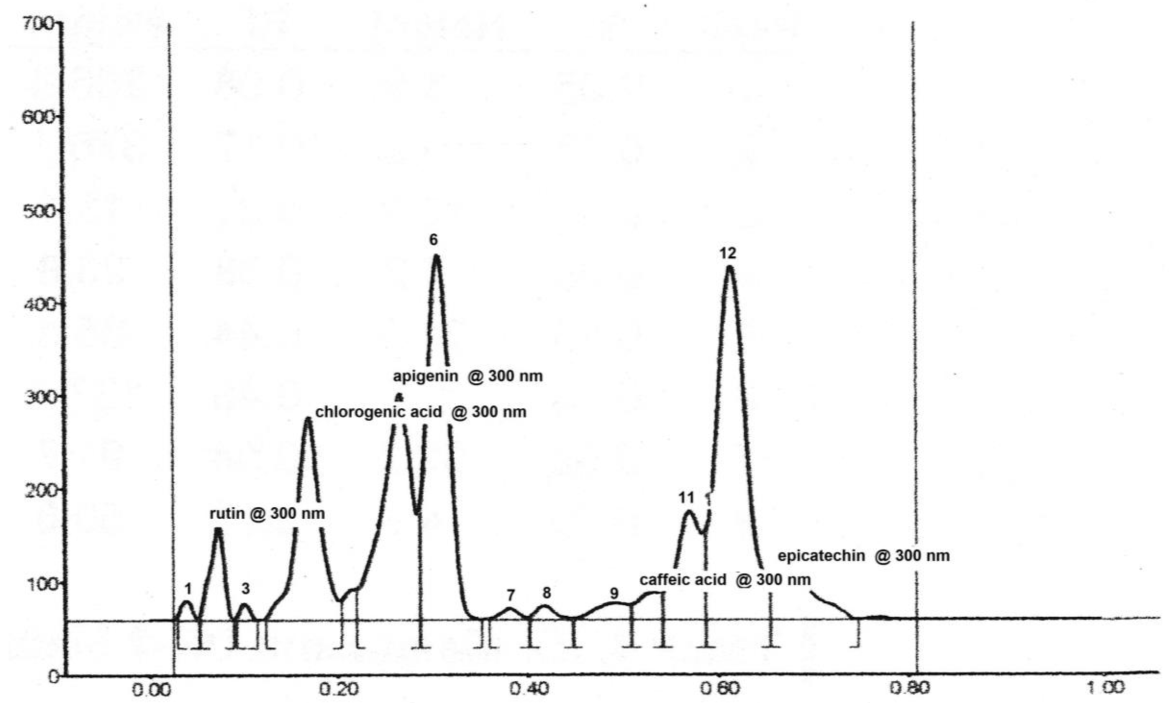

Peak	Start Rf	Start Height	Max Rf	Max Height	Max %	End Rf	End Height	Area	Area %	Assigned substance
1	0.03	1.0	0.04	20.6	1.29	0.05	1.1	249.4	0.57	unknown
2	0.05	2.1	0.07	100.9	6.31	0.09	0.8	1537.9	3.51	rutin
3	0.09	0.9	0.10	17.2	1.08	0.11	0.5	185.3	0.42	unknown
4	0.12	0.5	0.17	217.3	13.59	0.20	19.8	5724.7	13.07	chlorogenic acid
5	0.22	32.4	0.27	233.5	14.60	0.29	111.7	7573.2	17.30	apigenin
6	0.29	114.6	0.31	391.6	24.49	0.35	0.1	9577.4	21.87	unknown
7	0.36	1.1	0.38	11.7	0.73	0.40	2.3	225.0	0.51	unknown
8	0.40	2.5	0.42	14.6	0.92	0.45	1.4	293.6	0.67	unknown
9	0.45	1.4	0.49	17.7	1.10	0.51	15.5	596.7	1.36	unknown
10	0.51	15.6	0.53	28.7	1.80	0.54	27.8	670.7	1.53	caffeic acid
11	0.54	28.0	0.57	115.8	7.24	0.59	90.6	3004.8	6.86	unknown
12	0.59	91.5	0.61	378.7	23.68	0.65	49.1	11864.1	27.10	unknown
13	0.65	49.1	0.67	50.7	3.17	0.75	0.0	2282.8	5.21	epicatechin

Figure S3. HPLC-PDA (278 nm) chromatograms of DR2B and DR2C extracts.

A

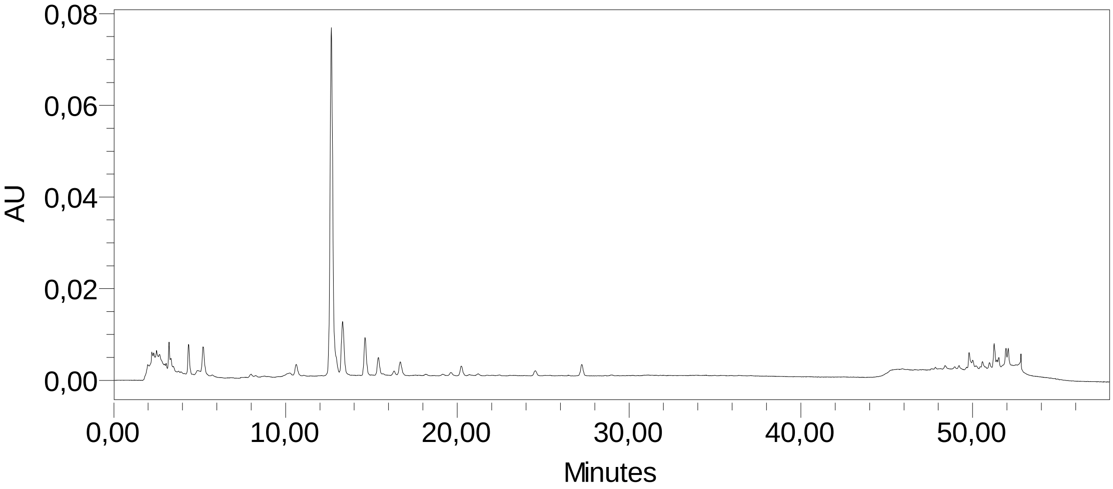

B

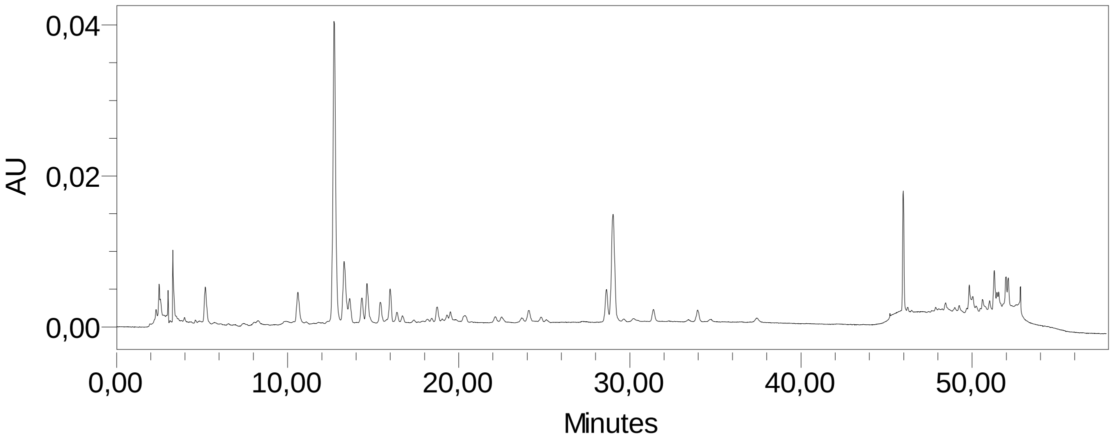

Figure S4. Effect of DR2B and DR2C extracts the viability and lactate secretion of Caco2 cells under normoxia and cobalt chloride ($CoCl_2$)-induced hypoxia. Cell viability expressed as % of control. Data are mean ± SE from two independent biological replicates, each one performed in at least three technical replicates (n = 2). ** $p < 0.01$ vs tBOOH by ANOVA followed by Dunnett's Multiple Comparison Post Test.

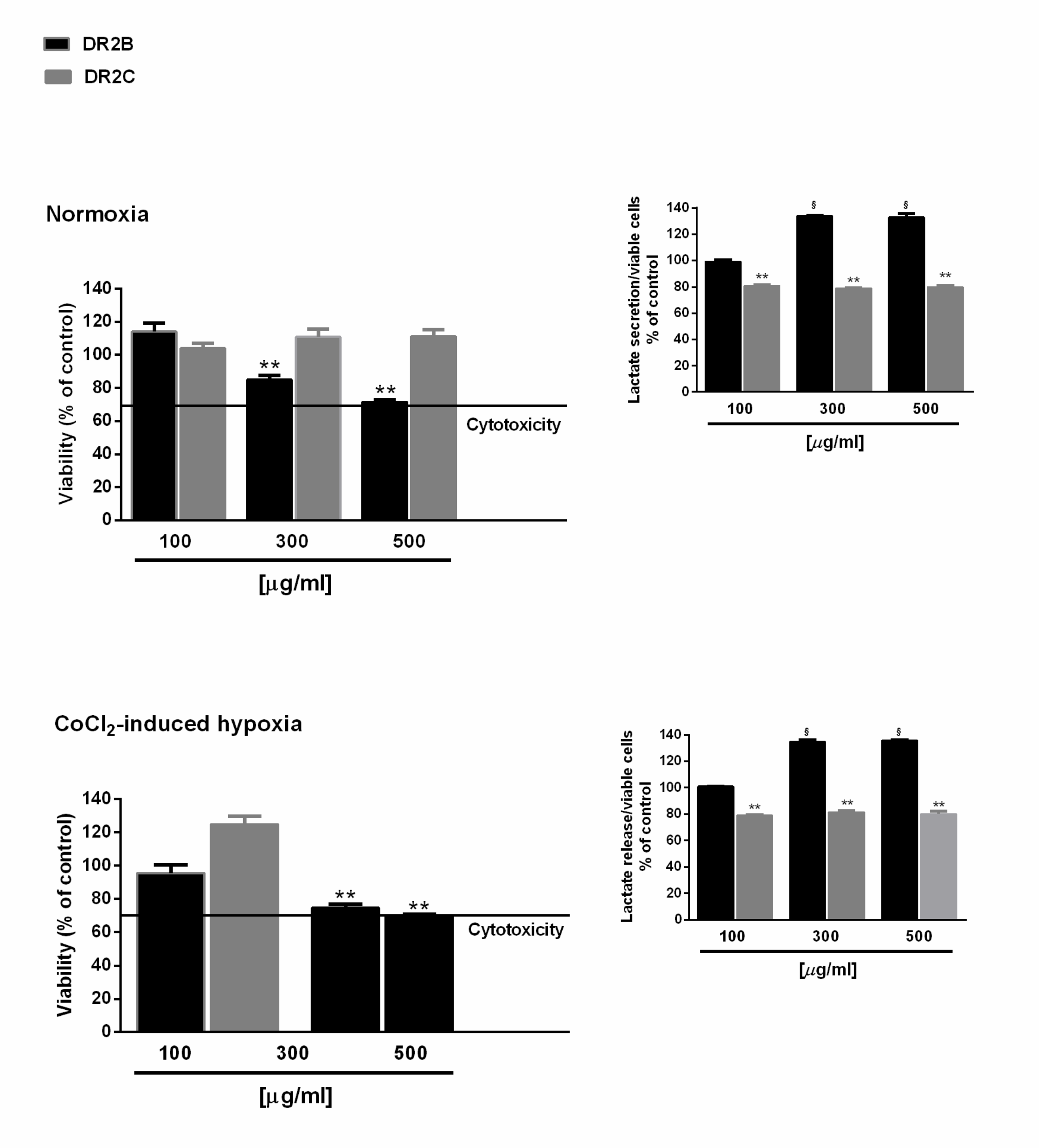

Supplement: Supplementary file 1 [file molecules-23-02066-s001.pdf]
